# Songbird nests on the ground as islands of diversity of ptyctimous mites (Acari: Oribatida) in the primeval Białowieża Forest (Poland)

**DOI:** 10.1007/s10493-023-00800-8

**Published:** 2023-07-13

**Authors:** Wojciech Niedbała, Marta Maziarz, Grzegorz Hebda, Tomasz Rutkowski, Agnieszka Napierała, Przemysław Kurek, Michał Zacharyasiewicz, Richard K. Broughton, Jerzy Błoszyk

**Affiliations:** 1 Natural History Collections, Faculty of Biology, Adam Mickiewicz University, Uniwersytetu Poznańskiego 6, Poznań, 61-614 Poland; 2 Museum and Institute of Zoology, Polish Academy of Sciences, Wilcza 64, Warsaw, 00-679 Poland; 3 Institute of Biology, University of Opole, Oleska 22, Opole, 45-040 Poland; 4 Department of General Zoology, Faculty of Biology, Adam Mickiewicz University, Uniwersytetu Poznańskiego 6, Poznań, 61-614 Poland; 5 Department of Plant Ecology and Protection of Environment, Faculty of Biology Adam, Mickiewicz University, Uniwersytetu Poznańskiego 6, Poznań, 61-614 Poland; 6 UK Centre for Ecology & Hydrology, Maclean Building, Benson Lane, Crowmarsh Gifford, Wallingford, OX10 8BB UK

**Keywords:** Biodiversity, Assemblage structure, Nest of birds, Ptyctimous mites, Unstable microhabitats

## Abstract

Due to specific microclimatic conditions and accumulation of organic matter, bird nests are microhabitats that are often inhabited by various invertebrates, including mites (Acari). We tested whether nests of the ground-nesting passerine *Phylloscopus sibilatrix* (Bechstein) (Passeriformes: Phylloscopidae) [wood warbler] were associated with an increased local diversity of ptyctimous mites (Acari: Oribatida) on the forest floor in the Białowieża National Park, East Poland. In 2019–2020, we analysed 150 warbler nests shortly after they had been vacated by the birds, and additionally we sampled mites in leaf litter at 1 and 6 m distances from 21 of the collected nests. We found on average more mite species in nests sampled in 2020 than in nests sampled in 2019. Although the species composition largely overlapped between bird nests and the litter, bird nests contained a greater average number of mite species than litter samples, including species found only – *Microtritia minima* (Berlese) and *Phthiracarus crenophilus* Willmann – or mostly – *Euphthiracarus cribrarius* (Berlese) and *Phthiracarus globosus* (C.L. Koch) – in bird nests. The results suggest that the presence of bird nests may increase the local diversity of the invertebrate species assemblage.

**Supplementary Information:**

The online version contains supplementary material available at 10.1007/s10493-023-00800-8.

## Introduction

Birds build their nests mainly to reproduce, but the nests can become microhabitats for other groups of animals, including a large diversity of invertebrates. Due to the diverse locations of bird nests (e.g., in tree tops, tree hollows, on the ground, or on the water), their varying structure (open, domed or enclosed) and their variable composition (Hansell and Overhill 2000; Deeming and Reynolds 2015), nests offer diverse microhabitats that provide a wide spectrum of conditions for their inhabitants (Woodroffe 1953; Klekowski and Opaliński 1986; Napierała et al. 2021). Bird nests can act as islands of distinctive microclimate with a specific temperature and humidity (Webb et al. 1998; Sinclair and Chown 2006; Dawson et al. 2011; Maziarz et al. 2020), accumulated organic matter, and potential food resources, such as dead plant matter, bird remains, feathers, faeces and detritus (Krištofík et al. 1993; Tryjanowski et al. 2001; Pilskog et al. 2014). As such, bird nests can attract specialist invertebrate species that may colonize such places, and consequently promote a high local diversity of the bird-nest associated fauna.

The invertebrate fauna inhabiting bird nests is represented by numerous taxonomic groups, which coexist with the host birds or use nests after they had been abandoned by the hosts. The most common invertebrate groups include spiders (Svatoň 1985; Heneberg 2018; Machač 2021), ants (Haemig 2001; Maziarz et al. 2018, 2021), beetles (Norman 1906; Watt 1980; Cosandey et al. 2021; Pushkin et al. 2021), moths (Nasu et al. 2012; Boyes 2018; Boyes and Lewis 2019), flies (Gold and Dahlsten 1983; Sabrosky et al. 1989; Hori et al. 1990; Eeva et al. 2015) and fleas (Harper et al. 1992; Heeb et al. 1996; Kędra et al. 1996). Mites (Acari) are also commonly recorded in bird nests, represented by many orders and families that occur in large numbers. However, due to their microscopic size and difficulty of species identification the community of mite species inhabiting bird nests still remains poorly understood.

Mite assemblages in bird nests comprise different ecological groups, including obligatory bird parasites (Philips 2000; Błoszyk et al. 2016), obligatory nidicoles that are not parasites (Chmielewski 1982; Fain et al. 1990, 1993; Solarz et al. 1998, 1999, 2004; Ardeshir 2010) and nidicoles, which are only indirectly associated with the host (Fenďa and Pinowski 1997; Krumpál et al. 1997; Fenďa et al. 1998; Gwiazdowicz 2003; Fenďa and Schniererová 2004; Błoszyk et al. 2005, 2006; Napierała et al. 2020). Mite assemblages that have been extensively examined in bird nests include Mesostigmata, e.g., Uropodina (Acari: Parasitiformes), which can be regarded as facultative nidicoles inhabiting bird nests (Krištofík et al. 2001, 2005, 2007; Mašán 2001; Gwiazdowicz and Mizera 2002; Fenďa and Schniererová 2004; Gwiazdowicz et al. 2005, 2006; Błoszyk et al. 2005, 2006, 2009; Bajerlein et al. 2006; Napierała et al. 2021). Other groups of mites which were found in bird nests are Oribatida (Błoszyk and Olszanowski 1985; Fain et al. 1993; Tryjanowski et al. 2001; Ardeshir 2010; Ermilov 2013; Lebedeva and Poltavskaya 2013; Meleschuk and Skilsky 2017; Melekhina et al. 2019; Napierała et al. 2021; Liu et al. 2022; Mangová et al. 2022, Laska et al. 2023) and Prostigmata (Kaźmierski 1996; Bochkov 2004; Bochkov and OConnor 2010; Skoracki et al. 2012; Kaźmierski et al. 2018; Laska et al. 2023). However, most of the publications on Oribatida in bird nests focus on faunistic studies, without any analysis of the examined communities (Błoszyk and Olszanowski 1985; Fain et al. 1993; Tryjanowski et al. 2001; Lebedeva and Poltavskaya 2013; Meleschuk and Skilsky 2017; Melekhina et al. 2019; Liu et al. 2022). As such, it remains unclear whether the assemblage of mites inhabiting bird nests differ from the surrounding habitat, and whether some species occur in bird nests more often than elsewhere.

Ptyctimous mites (Acari: Oribatida) are one of the best studied groups of free-living mites (Niedbała and Liu 2023), but information on their biology and the habitat preferences of individual species is fragmentary. Ptyctimous mites are macrophytophages at all life stages and feed on plant debris. They play an important role in the processes of mechanical fragmentation and chemical modification of the organic debris in which they live (Niedbała 2008). The research conducted so far has focused mainly on taxonomic issues and the zoogeography of this group of mites described in monographs (Niedbała 2000, 2001, 2002, 2004, 2006, 2011) and the recently published world catalogue of these mites (Niedbała and Liu 2023). The exceptions are two studies describing assemblages of ptyctimous mites in Australia (Niedbała and Szywilewska-Szczykutowicz 2017; Niedbała et al. 2021), and one work focusing on the communities of ptyctimous mites inhabiting soil and dead wood in the Białowieża Forest (Niedbała et al. 2020). There are also two studies concerning mite assemblages in bird nests that include ptyctimous mites (Mangová et al. 2022; Laska et al. 2023), including one outlining the assemblages of oribatid mites in bird’s nests in an urban environment, and another describing mite assemblages (including representatives of Mesostigmata, Trombidiformes and Sarcoptiformes) inhabiting nests of wood warblers (*Phylloscopus sibilatrix*) collected in the Wielkopolska National Park (W Poland). However, information on the assemblage of ptyctimous mites within bird nests situated on the forest floor is still rare, and it remains unclear whether bird nests might represent islands of increased diversity of the mite assemblage in forest ecosystems.

Our study is one of the first to document the ptyctimous mite assemblage from bird nests situated on the forest floor in pristine habitat, specifically the nests of wood warblers (see also Laska et al. 2023), collected from the best-preserved, old-growth stands of the Białowieża Forest. The wood warbler is an insectivorous songbird (body weight ca. 10 g) that winters in equatorial Africa and breeds in temperate European forests (Cramp 1992). The breeding numbers of wood warblers have declined across Europe, but the species remains of least concern (Maag et al. 2022; BirdLife International 2023). The breeding season of wood warblers begins in late April and ends in July-August when nestlings from replacement clutches (after initial loss) or second broods leave the nest. The typical clutch size in Białowieża National Park is 5–7 eggs, and the nestling stage lasts 12–13 days (Cramp 1992; Wesołowski and Maziarz 2009).

**Fig. 1:**
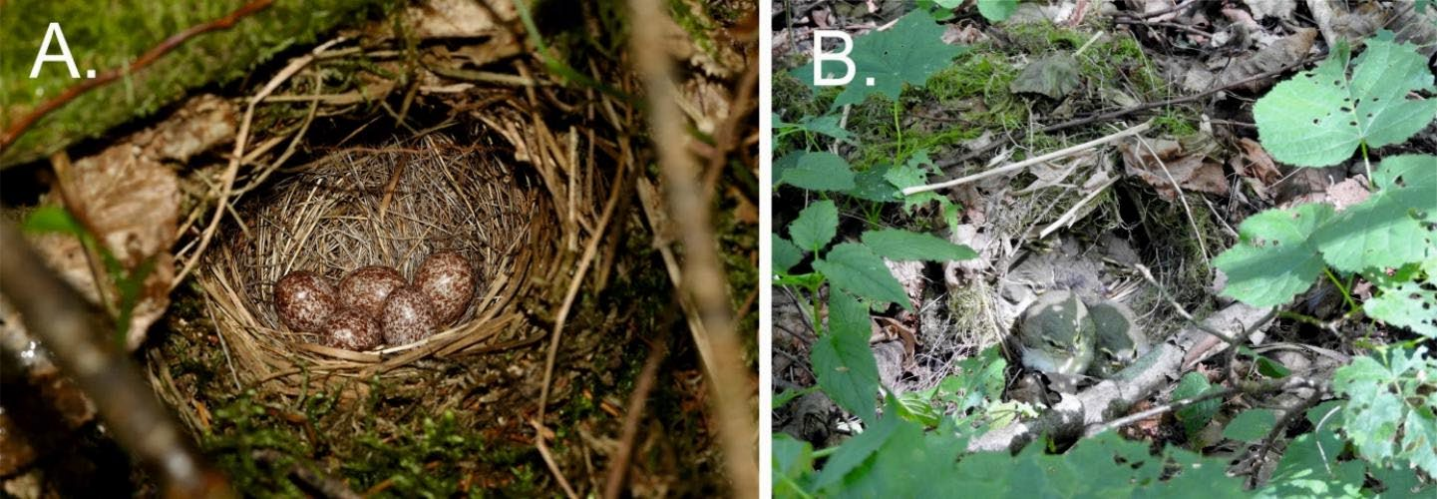
Nests of wood warblers, *Phylloscopus sibilatrix*, on the forest floor, with (**A**) eggs and (**B**) nestlings in the Białowieża Forest, Poland (photos by G. Hebda and M. Maziarz)

The nests of wood warblers are dome-shaped and always situated directly on the ground, usually nearby a tussock of vegetation or fallen dead wood (Wesołowski 1985; Cramp 1992; Maziarz et al. 2021) (Fig. 1). The nests are composed mainly of grass blades and tree leaves with an admixture of mosses (Napierała et al. 2021). Wood warblers build new nests at each breeding attempt, so these are specific yet ephemeral microhabitats on the forest floor, used for a short period of time.

The aims of this study were to test whether: (1) ptyctimous mite assemblages within bird nests differ inter-annually, taking into account inter-breeding spatial changes of the nest location, and (2) accumulated plant material on the ground in the form of a songbird nest is a focal point for ptyctimous mite diversity. As bird nests can attract some species, like obligatory bird parasites and nidicoles (see above), we hypothesized that the presence of bird nests might increase the local diversity of ptyctimous mites in comparison to the surrounding soil and leaf litter in the forest ecosystem.

## Materials and methods

### Study area

The research study was conducted in 2019–2020, mainly within the study plots (46–200 ha each), which were distributed across the best-preserved part of the Białowieża Forest (eastern Poland), strictly protected within the Białowieża National Park (BNP; coordinates of Białowieża village: 52°42′N, 23°52′E). However, a few observations were conducted outside of the study plots and in managed deciduous stands, adjacent to the BNP. The observations were performed in accordance with the relevant guidelines and regulations of the BNP, and were permitted by the Ministry of the Environment in Poland (DOP-WPN.286.11.2018.MD).

The Białowieża Forest is an exemplar forest of the temperate climate zone in lowland Europe (Tomiałojć 1984; Faliński 1986; Jaroszewicz 2019). The strictly protected stands are a relic of the mixed-coniferous and deciduous primeval forests that once covered European lowlands before deforestation and transformation by humans. The pristine features of the old-growth stands in BNP include a multi-species and multi-storey structure consisting of trees of different ages (up to a few hundred years old), and many standing and fallen dead trees, as well as a high overall species diversity (Tomiałojć 1984; Tomiałojć and Wesołowski 2004).

The deciduous stands (*Tilio-Carpinetum*), which dominate in BNP, are mainly formed by hornbeams (*Carpinus betulus* L.), limes (*Tilia cordata* Miller), and oaks (*Quercus robur* L.), with an admixture of other tree species, including maples (*Acer platanoides* L.) and spruces (*Picea abies* (L.) H. Karst.) (Faliński 1986). The most dominant tree species in the mixed-coniferous (*Pino-Quercetum*) stands are spruces, pines (*Pinus sylvestris* L.) and oaks, with less abundant birches (*Betula* spp.), but there is also an increasing number of hornbeams and limes in recent decades (Wesołowski et al. 2015). The adjacent tree stands of the commercial forests have a more uniform structure than those in BNP, with fewer tree species of a younger age, and also less dead wood. The most common tree species are hornbeams and oaks, with less frequent birches, aspens (*Populus tremula* L.), and spruce trees. For further description of the study areas see Broughton et al. (2020) and Napierała et al. (2021).

### Data collection

In 2019–2020, we analysed 150 wood warbler nests (69 nests in 2019 and 81 nests in 2020), which were collected between May and June within a few days of the nestlings leaving the nest (fledged), or the breeding attempts having failed naturally but the nest structure remained intact. Overall, 87% of nests in both years were distributed at least 50 m apart (the range of nearest-neighbor distances was 6-899 m in either year), mostly within the study plots in BNP (115 of 150 nests). The study plots were surveyed intensively to locate all wood warbler nests from 2018 (Broughton et al. 2020). The nests were built by birds in different locations within the plots every year, and each nest was examined once, representing an independent sample.

We also tested whether the assemblage structure of ptyctimous mites was more diverse in the wood warbler nests compared to the surrounding habitat on the forest floor. Therefore, in 2020, we also collected samples of leaf litter and small woody debris covering the soil on the forest floor (hereafter ‘litter’). The litter was collected from an area corresponding to the average size of a wood warbler nest (i.e., within a circe of ca. 15 cm diameter) and of a volume aproximate to bird nests. The litter was sampled on the same transect line with a random direction, at distances of 1 m (hereafter: Ref 1) and 6 m (hereafter: Ref 6) from each of the 21 wood warbler nests (n = 63 samples in total, including bird nests). The 21 nests were randomly chosen out of the 150 nests analyzed (see above). The collected nests and the litter samples were placed into separate sealed and labelled plastic bags.

All samples were transported from the forest and then stored in a fridge (ca. 1–5 °C) for up to 6 days before the invertebrate extraction. Mite specimens were extracted with Tullgren funnels for 2–3 days, which was enough time to dry out the light and loose material of leaf litter and bird nests and extract the specimens from them. The extracted specimens were then preserved in 70–80% ethanol. The mite specimens were sorted out from the samples and then identified with an Olympus BX51 stereoscopic microscope, after they had been cleared in 80% lactic acid. Species identification was carried out by the first author (Wojciech Niedbała), and the material has been deposited in the Natural History Collections at the Faculty of Biology of the Adam Mickiewicz University in Poznań (https://amunatcoll.pl/).

### Data analysis

As the ptyctimous mite assemblages within nests of the wood warblers might vary from year to year, the inter-annual differences in the species richness were tested with a one-way ANOVA. We treated the nests as independent samples, as they were presumably built by different females (considering the low return rate of wood warblers of 5% in Białowieża Forest; Wesołowski et al. 2009), and used by breeding birds only once. To compare the ptyctimous mite assemblage between the nests and the corresponding litter samples, a one-way repeated-measures ANOVA was used. As we do not have complete data from litter from the first year of the research, both analyses were conducted separately, using R v.3.4.3 (R Core Team 2021). Where necessary, and prior to analyses, skewed data were transformed with logarithmic or exponential functions to obtain a normal or at least symmetrical distribution. Post-hoc analysis was done by a Tukey test using the ‘glht’ function with the ‘multcomp’ package (Hothorn et al. 2008). Very high skewness of zero-inflated data, and their high inter-annual variability, meant that the raw data of abundance were used only in ordination analyzes.

The occurrence of some ptyctimous mite species may be limited to some preferred habitat types, and occur only or mostly within them. Therefore, the presence of those species would indicate the specificity of a given microhabitat against the background of the environment. To check which ptyctimous mite species from the analyzed assemblages were a good indicator for the sample type (nest or reference samples), an indicator species analysis was performed (Dufrêne and Legendre 1997). According to this method, for each species the indicator value was calculated as the product of the relative frequency (the proportion of sites of type *j* with species *i*) and relative average abundance in clusters (the proportion of the number of individuals of species *i* that are in a *j* type of site) multiplied by 100. The calculations were done with R v.3.4.3 using the ‘indval’ function in the ‘labdsv’ package (Roberts 2012).

Depending on the defined environmental conditions, mites may form variably dispersed and overlapping clusters of species or samples. Our samples were divided into two datasets depending on year (inter-annual variation) or location, i.e., from a nest or a reference sample of litter (reflecting spatial variation with nests as potential biodiversity hot-spots). To assess correlations between assemblages/clusters presented as different datasets in time and on the spatial gradient, a canonical correspondence analysis (CCA) was performed with CANOCO v.5 software (Šmilauer and Lepš 2014). The aim of the ordination analysis is to arrange the samples in such a way that those with a similar species composition are located close to each other on the axes, and different samples are distant from each other.

## Results

### Assemblages of ptyctimous mites in wood warbler nests

**Fig. 2:**
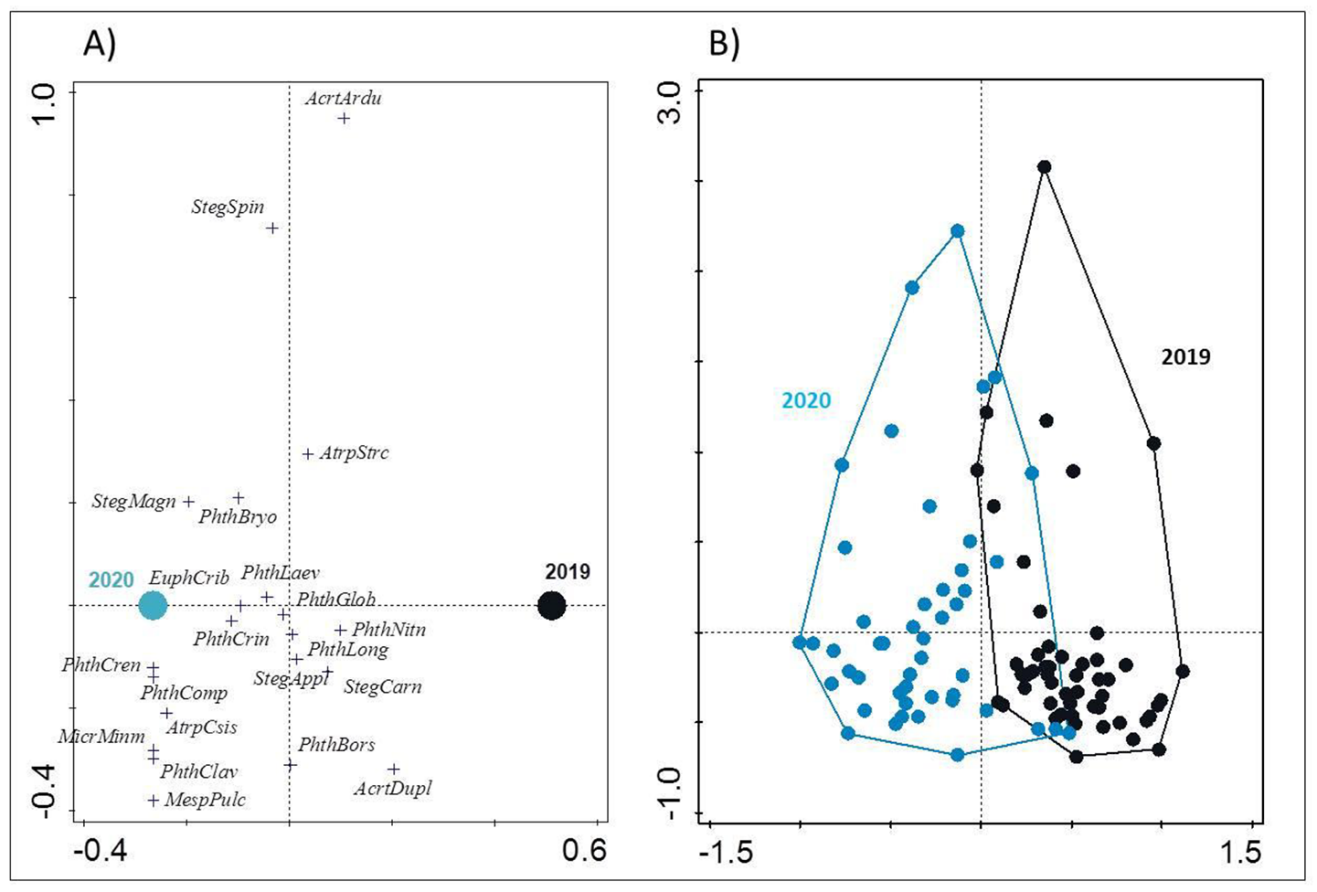
Canonical correspondence analysis (CCA) scatterplot comparing the assemblages of ptyctimous mites recorded in wood warbler nests collected in 2019 (n = 69) and 2020 (n = 81) in Białowieża Forest, showing ordination of (**A**) species based on the nests they were found in, and (**B**) nests based on their species composition. First and second axes explained 16.71% of variation in ptyctimous mite assemblages. On the left panel, the dots marked 2019 and 2020 are centroids

Wood warbler nests contained from 1 to 10 species of ptyctimous mites, and in most cases (66%) it was 4–7 species per nest. The mean (± SD) number of species from nests differed between years with 4.4 ± 2.1 species recorded in 2019 (a total 17 species of 2,464 specimens; n = 69 nests) and 6.7 ± 1.8 in 2020 (a total 20 species of 4,642 specimens; n = 81 nests; one-way ANOVA: *F*_1,148_ = 52.3, *P* < 0.001). All species recorded in 2019–2020 in the wood warbler nests (n = 150) are listed in Table S1. Moreover, CCA analysis confirmed inter-annual differences in the ptyctimous mite assemblages (Fig. 2). The qualitative differences in inter-annual assemblages of ptyctimous mites were visible by well separated assemblage samples for each year (Fig. 2B).

### Ptyctimous mite assemblages in wood warbler nests compared to adjacent soil and litter

In 2020, a total of 20 species of ptyctimous mites (1,448 specimens) were recorded in 21 warbler nests, including *Phthiracarus crenophilus* Willmann, a new species for the Białowieża Forest (Table 1). Meanwhile, the Ref 1 and Ref 6 litter samples that were collected at a respective 1 and 6 m from the nests contained 18 ptyctimous mite species in total (1,533 specimens) (Table 1). According to the indicator species analysis, *Euphthiracarus cribrarius* (Berlese) and *Phthiracarus globosus* (C.L. Koch) were much more likely to be associated with warbler nests than with nearby litter (Table 1). In contrast, none of the mite species were significantly associated only with the litter samples.

**Table 1:** The frequency and indicator values for the ptyctimous mite species across the 63 samples (n) of the wood warbler nests and litter samples: 1 m (Ref 1) and 6 m (Ref 6) from each bird nest in 2020. Indicator values differed significantly (*P* < 0.01) between samples only for *Euphthiracarus cribrarius* and *Phthiracarus globosus* (n.s., *P* > 0.05). Indicator values are derived from indicator species analysis (see Methods), where higher values reflect a stronger association with the sample type (Nest, Ref 1 or Ref 6)

Species	Freq	Indicator values			*P*
		Nest (n = 21)	Ref 1 (n = 21)	Ref 6 (n = 21)	
*Euphthiracarus cribrarius* (Berlese)	32	64.7	3.7	3.5	0.001
*Phthiracarus globosus* (C.L. Koch)**	22	37.9	1.6	6.4	0.009
*Steganacarus* (*T.*) *carinatus* (C.L. Koch)	44	37.1	18.5	13.1	n.s.
*Phthiracarus nitens* (Nicolet)	28	30.2	18.5	2.2	n.s.
*Phthiracarus longulus* (C.L. Koch)	41	25.5	24.6	15.7	n.s.
*Phthiracarus crinitus* (C.L. Koch)**	17	23.8	7.7	1.5	n.s.
*Phthiracarus bryobius* Jacot**	13	22.1	7.5	0.1	n.s.
*Steganacarus* (*S.*) *applicatus* (Sellnick)	43	19.2	28.6	20.1	n.s.
*Phthiracarus laevigatus* (C.L. Koch)*,**	10	18.4	0.2	3.5	n.s.
*Phthiracarus compressus* Jacot**	16	16.5	6.1	4.4	n.s.
*Microtritia minima* (Berlese)*	2	9.5	0.0	0.0	n.s.
*Phthiracarus boresetosus* Jacot**	9	8.6	2.4	4.3	n.s.
*Atropacarus* (*A.*) *striculus* (C.L. Koch)	17	8.1	3.0	17.1	n.s.
*Steganacarus* (*S.*) *magnus* (Nicolet)*,**	11	5.4	4.4	7.0	n.s.
*Phthiracarus crenophilus* Willmann*,***	1	4.8	0.0	0.0	n.s.
*Acrotritia duplicata* (Grandjean)	7	4.5	7.1	0.9	n.s.
*Atropacarus* (*A.*) *csiszarae* (Balogh et Mahunka)**	11	2.4	8.1	5.3	n.s.
*Acrotritia ardua* (C.L. Koch)**	3	2.3	1.7	0.8	n.s.
*Steganacarus* (*S.*) *spinosus* (Sellnick)*,**	4	1.5	0.8	3.2	n.s.
*Phthiracarus clavatus* Parry**	8	0.3	7.0	8.3	n.s.

*rare and sparse species in Poland (Niedbała 2008); ** rare and sparse species in Białowieża Forest (Niedbała et al. 2020), ***new species in Białowieża Forest

**Fig. 3:**
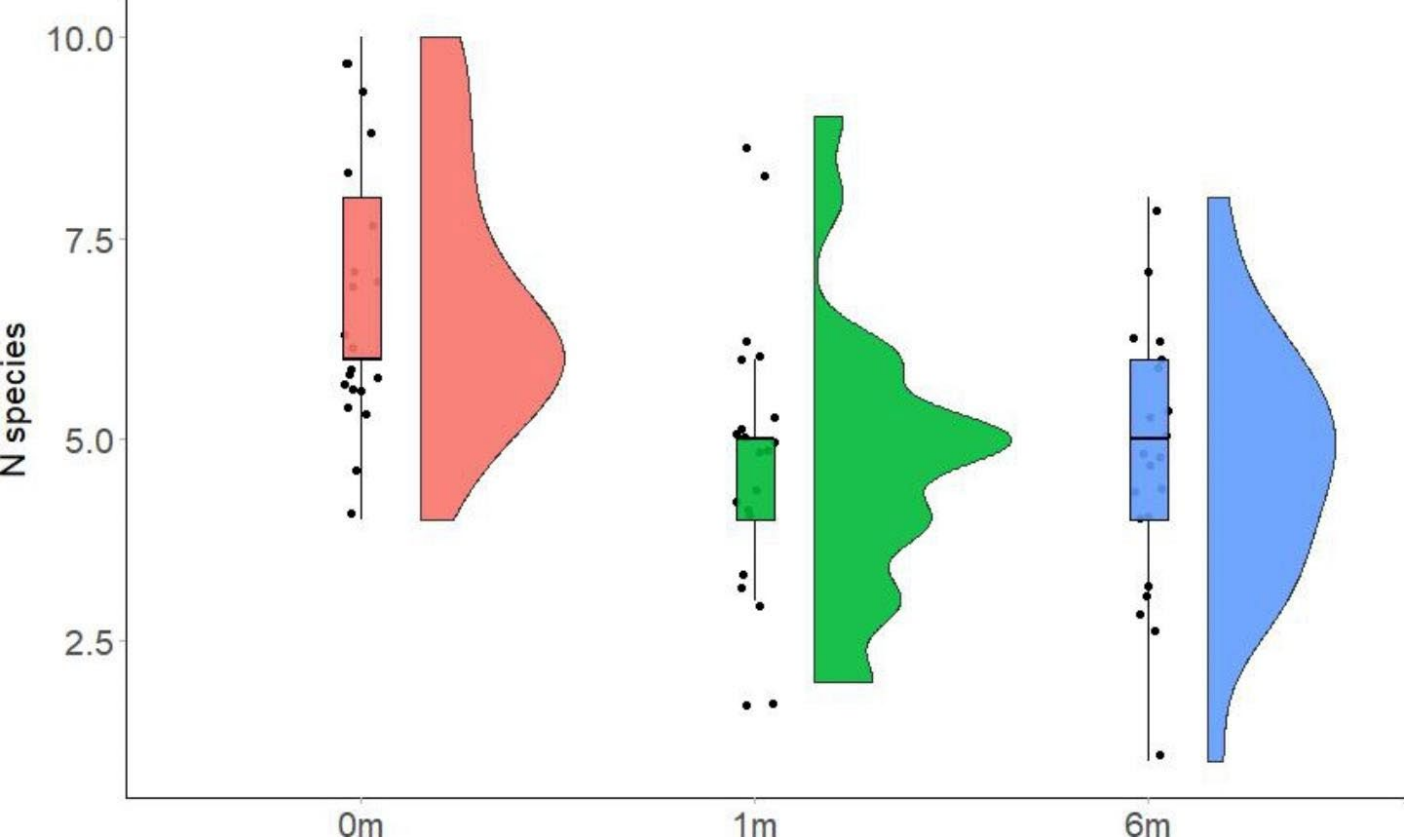
Plots showing that wood warbler nests (n = 21) have a higher number of ptyctimous mite species than litter at distances of 1 m (Ref 1, n = 21) and 6 m (Ref 6, n = 21) from nests. Plots show the individual observations (points), the lower and upper limits of boxplots that correspond to the first and third quartiles, and their probability distribution

The mean (± SD) number of species was significantly higher in wood warbler nests (6.8 ± 1.7) than in the litter samples at distance of 1 m (Ref 1: 4.7 ± 1.7; t = -3.98, *P* = 0.0006) and 6 m (Ref 6: 4.7 ± 1.6; t = -4.07, *P* = 0.0004) from the nest. There was no significant difference in the number of species between litter samples Ref 1 and Ref 6 (t = -0.09, *P* > 0.05; Fig. 3).

**Fig. 4:**
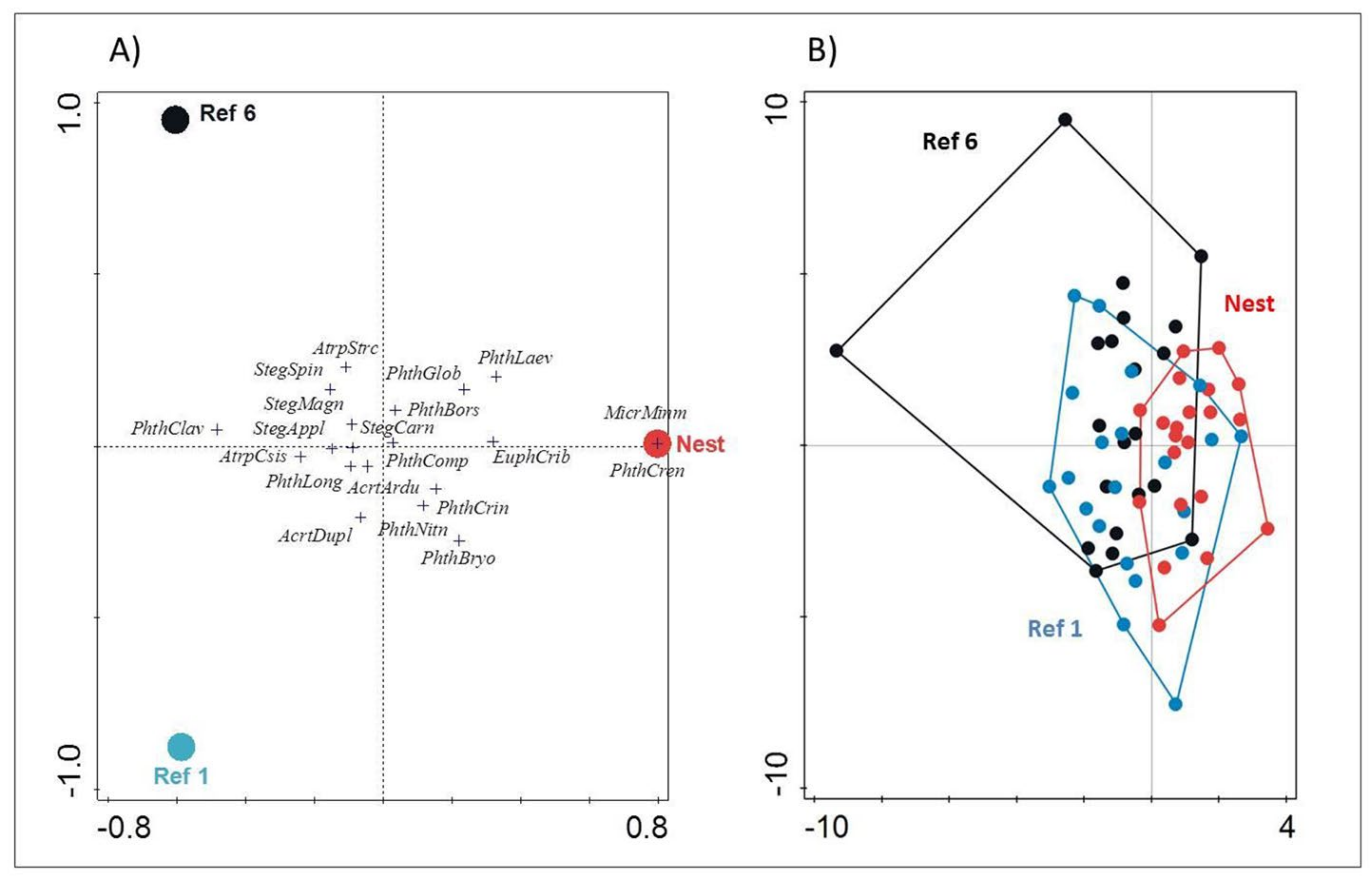
Canonical correspondence analysis (CCA) scatterplot showing the relationships between the assemblage of ptyctimous mites found in 21 nests of the wood warbler (Nest) and respective litter samples collected at 1 m (Ref 1) and 6 m (Ref 6) distances from nests (n = 21 each) in Białowieża Forest. Ordination of (**A**) species based on the samples they were found in, and (**B**) samples based on their species composition. First and second axes explained 3.62% of the variation in ptyctimous mite communities. On the left panel the dots are centroids

In contrast to the indicator analysis in Table 1, the CCA analysis showed broader qualitative differences between warbler nests and litter samples of Ref 1 and Ref 6 (Fig. 4). The communities of ptyctimous mites in warbler nests were characterized by two species – *Microtritia minima* (Berlese) and *P. crenophilus*, which occurred only in the nests, and a further few species that occurred more often in the nests than in the litter, such as *E. cribrarius*, *P. globosus* and *Phthiracarus laevigatus* (C.L. Koch) (Fig. 4A). Other species, which are scattered in the middle of the axes in Fig. 4A, formed the core of the mite assemblage and were common both in the nests and litter samples. Nevertheless, species composition appeared to be more consistent within the bird nests than in the litter samples, as ptyctimous mites formed a more consolidated group in nests, but were more widely scattered in litter samples (Fig. 4B).

## Discussion

We found that the species diversity of ptyctimous mites was higher in wood warbler nests on the forest floor than in reference samples of leaf litter at different distances from the nests. These results support the hypothesis that the presence of bird nests is associated with an increased local diversity of ptyctimous mites. The statistical difference between mite assemblages in bird nests and in litter was due to a few species found only (*M. minima* and *P. crenophilus*) or mostly (*E. cribrarius* and *P. globosus*) in the warbler nests, as all of the other species recorded in litter also occurred within bird nests (Table 1). However, there were no significant differences in species composition between litter samples collected at 1 and 6 m distances from the nests (Fig. 4B), indicating that the effect of nests on the ptyctimous mite assemblage was very limited in spatial scale, to < 1 m.

The large overlap in the composition of ptyctimous mite species between wood warbler nests and litter is likely related to the nest location on the ground, which would promote nest colonisation by eurytopic soil mites, including ptyctimous mites. Soil saprophagous mites could also be transported on the nest material by the birds, such as when collecting moss, grass blades or tree leaves collected from the forest floor when building their nests. Thus, the accumulation of organic matter in the form of wood warbler nests might promote higher biodiversity of ptyctimous mites than in leaf litter nearby. Yet, because little is known about the habitat preferences of ptyctimous mites, distinguishing the typical specialised nidicoles among the species recorded in wood warbler nests is currently challenging. However, the fact that some species (*M. minima*, *P. crenophilus*, *E. cribrarius*, *P. globosus*) were largely or wholly restricted to nests, rather than the surrounding leaf litter, may indicate some degree of specialisation to nest habitats.

Bird nests have been shown to provide nest-dwelling invertebrates of various taxa with a food source, such as for mites which feed on keratin from bird skin or feathers (Chmielewski 1982; Fain et al. 1990; Solarz et al. 1998, 1999, 2004), or a suitable microclimate (Krištofík et al. 1993; Tryjanowski et al. 2001; Pilskog et al. 2014). The active nests of wood warblers, warmed by the owners’ body heat, provides an advantageous microclimate for the development of *Myrmica* and *Lasius* ant larvae or pupae, attracting ants to relocate their offspring into bird nests (Maziarz et al. 2020, 2021). However, due to limited knowledge of the biology of ptyctimous mites, it is not clear what specific conditions typical of bird nests may attract the mites to colonise them. Nevertheless, the findings confirmed that bird nests could contribute to increasing the local diversity of mite species, as previously highlighted for other invertebrates (Watt 1980; Svatoň 1985; Haemig 2001; Maziarz et al. 2018, 2021; Boyes and Lewis 2019; Cosandey et al. 2021).

The species richness of ptyctimous mites differed between years, being generally higher in 2020 than in 2019, including four species undetected in the latter. A similar situation of annual variation has been observed in Uropodina mite assemblages in the Białowieża Forest (J. Błoszyk, in prep.). The reasons for the inter-annual differences may be due to varying locations of wood warbler nests between years. The recording of new species in a subsequent year could suggest that the list of ptyctimous mite species is incomplete. Alternatively, the analysed nest and litter material did not contain any mite species that were previously found in dead wood in other areas of the Białowieża Forest e.g., *Protoribotria oligotricha* (Märkel) and *Phthiracarus opacus* (Niedbała) (Niedbała et al. 2020). Thus, long-term research that considers various micro-environments, including bird nests, would be valuable in revealing a more comprehensive picture of the species diversity for the region, particularly for the well-preserved, near-primeval stands of the Białowieża Forest.

The generally high species diversity of mite assemblages observed in the nests of wood warblers, and also in the nearby litter, could be related to the high degree of naturalness of the strictly protected forest in the BNP, where the research was mainly conducted (Jaroszewicz et al. 2019). The presence of a variety of microhabitats and large amounts of dead wood, which is rare in heavily managed woods (Bobiec 2002), could enable many species from different taxonomic groups to find suitable habitats in a small area, resulting in a high local (alpha) diversity (Gwiazdowicz 1998, 1999; Gwiazdowicz et al. 1999; Błoszyk and Olszanowski 1999; Tomiałojć and Wesołowski 2004; Napierała et al. 2020; Niedbała et al. 2020). However, the geographical location of the Białowieża Forest could also facilitate a higher regional (gamma) diversity of animal communities, including mites (Błoszyk et al. 2003; Tomiałojć and Wesołowski 2004; Błoszyk and Napierała 2021).

In conclusion, our study assesses the ptyctimous mite assemblage inhabiting bird nests in an undisturbed forest ecosystem. It is one of the first studies documenting the assemblage of ptyctimous mites in bird nests that are situated on the forest floor, following the work by Napierała et al. (2021) and Laska et al. (2023), and the first one that includes comparison with litter in vicinity of the nests. In total, the 20 species of ptyctimous mites in wood warbler nests constituted ca. 50% of all of the ptyctimous mite fauna occurring in Poland (Niedbała 2008). Notably, almost three-quarters of the ptyctimous mite species found in warbler nests were relatively rare species in Poland (Table 1) and one (*P. crenophilus*) was a new species for the Białowieża Forest (compare with Niedbała et al. 2020).

In the examined nests we found a higher number of species of ptyctimous mites, which suggests an important role of bird nests for increasing local diversity of ptyctimous mites. Our research broadens the knowledge of the role of ground-nesting birds and their nests in forest ecosystems as hotspots of local biodiversity, which may have wider relevance for the presence of bird nests in other forests and habitats. In this context, the Białowieża Forest is a key point of reference for subsequent studies in Europe, especially in the light of threats to this ecosystem and the rapidly progressing and widespread changes in natural environments.

## Electronic supplementary material

Below is the link to the electronic supplementary material.

Supplementary Material 1

## Data Availability

The data presented in this study stored in a computer database called AMUNATCOLL and openly available at: https://amunatcoll.pl/.
