# Supplementary material for: Songbird nests on the ground as islands of diversity of ptyctimous mites (Acari: Oribatida) in the primeval Białowieża Forest (Poland)
Source: Exp Appl Acarol. 2023 Jul 13;90(3-4):169–84. doi: 10.1007/s10493-023-00800-8 (PMC10406712; doi:10.1007/s10493-023-00800-8)
Supplement: Supplementary file 1 — Supplementary Material 1 [file 10493_2023_800_MOESM1_ESM.docx]

**Table S1** Number of ptyctimous mites found in 150 nests.

| **Species** | **Total number of specimens** | **Nests (out of 150)** |
| --- | --- | --- |
| *S.* (*T.*) *carinatus* | 2507 | 108 |
| *P. globosus* | 873 | 98 |
| *P. nitens* | 570 | 116 |
| *E. cribrarius* | 508 | 81 |
| *P. longulus* | 467 | 98 |
| *S.* (*S.*) *spinosus* | 448 | 21 |
| *A.* (*A*.) *striculus* | 440 | 51 |
| *S.* (*S*.) *applicatus* | 410 | 78 |
| *P. laevigatus* | 146 | 31 |
| *P. crinitus* | 128 | 40 |
| *A. ardua* | 125 | 10 |
| *A.* (*A.*) *csiszarae* | 118 | 11 |
| *P. bryobius* | 106 | 37 |
| *P. compressus* | 97 | 16 |
| *S.* (*S.*) *magnus* | 36 | 12 |
| *M. minima* | 34 | 3 |
| *P. boresetosus* | 28 | 13 |
| *A. duplicata* | 27 | 12 |
| *P. crenophilus* | 26 | 6 |
| *P. clavatus* | 10 | 3 |
| *M*. (*P.*) *pulchra* | 2 | 1 |
